# Study on the species spectrum, drug resistance spectrum and molecular characteristics of clinical *Mycobacterium* isolates from three provinces in China

**DOI:** 10.3389/fmicb.2026.1782484

**Published:** 2026-05-29

**Authors:** Xinyue He, Ruihuan Wang, Haijia Zou, Machao Li, Xiuqin Zhao, Yi Guo, Yue Li, Dongfang Xu, Haican Liu, Mingxiang Huang, Xundi Bao, Yunhong Tan

**Affiliations:** 1 National Key Laboratory of Intelligent Tracking and Forecasting for Infectious Diseases, National Institute for Communicable Disease Control and Prevention, Chinese Center for Disease Control and Prevention, Beijing, China; 2 Anhui Chest Hospital, Hefei, China; 3 Anhui Provincial Center for Disease Control and Prevention, Hefei, China; 4 Fuzhou Pulmonary Hospital of Fujian, Fuzhou, China; 5 Hunan Province Chest Hospital, Changsha, China

**Keywords:** drug resistance, *Mycobacterium*, *Mycobacterium tuberculosis*, species identification, WGS

## Abstract

This study aimed to identify species identification, molecular typing, and drug resistance characterization of clinically isolated mycobacteria from Anhui, Fujian, and Hunan provinces, and to evaluate the value of whole-genome sequencing (WGS) in predicting drug resistance. WGS and phenotypic drug susceptibility testing (DST) were performed on clinical *Mycobacterium* isolates from those provinces. Bioinformatics analysis and chi-square test were used to compare inter-provincial differences in drug resistance and lineages. Of the 1,383 isolates, 95.30% belonged to the MTBC and 3.40% were NTM. Lineage 2 (74.07%) and lineage 4 (24.94%) were dominant in MTBC. The overall drug resistance rate was 32.08%, while MDR rate was 11.14%. Hunan had significantly higher MDR and RIF resistance rates than the other provinces (*p* < 0.05). INH had the highest resistance rate (19.37%). Fluoroquinolones exhibited significant inter-provincial differences in resistance rates. WGS exhibited >85% sensitivity and >96% specificity for first-line drug resistance prediction, and >77% sensitivity and >97% specificity for second-line drug resistance. Additionally, 87.92% of isolates showed consistent genotypic and phenotypic DST results. MTBC isolates exhibited pronounced inter-provincial variation in lineage composition. Geographical differences existed in drug resistance, particularly for MDR and RIF resistance. WGS was reliable for predicting first-line drug resistance, but unknown resistance mechanisms were detected, requiring further exploration to optimize prediction.

## Introduction

Tuberculosis (TB) remains a major worldwide infectious disease threatening public health. According to the WHO Global Tuberculosis Report, an estimated 10.7 million people worldwide developed TB in 2024 (World Health Organization, 2025). Drug-resistant tuberculosis (DR-TB), especially multidrug-resistant/rifampicin-resistant tuberculosis (MDR/RR-TB), further exacerbates the burden of prevention and control. This is owing to its long treatment duration, high cost, and low treatment success rate. The TB epidemic in China is particularly severe. It is characterized by the so-called “three highs and one low (Ding et al., 2020)” pattern. This pattern includes high incidence (696,000 new cases in 2024, ranking fourth globally), high infection rate [approximately 250–300 million latent tuberculosis infections (LTIs)], high drug resistance (estimated 28,000 MDR/RR-TB patients), and low treatment coverage (the estimated TB treatment coverage among the 0–14 age group ranks fourth from the bottom among high-burden countries; World Health Organization, 2025).

Furthermore, the reported incidence of non-tuberculous mycobacteria (NTM) is gradually increasing. NTM shares similar clinical manifestations and culture conditions with the *Mycobacterium tuberculosis* complex (MTBC; Marras and Daley, 2002; Tortoli, 2009). Notably, rapidly growing NTM is naturally resistant to some antibiotics, particularly first- and second-line anti-TB drugs (Stout et al., 2016; Hosseinali et al., 2023; Kwon et al., 2019). Misdiagnosis of NTM disease as pulmonary tuberculosis not only compromises therapeutic efficacy but may also lead to erroneous classification as multidrug-resistant (MDR) or rifampicin-resistant (RR) TB.

Compared with phenotypic DST, which requires *in vitro* cultivation, WGS offers a faster, safer, and more comprehensive solution. It can be used for bacterial species identification, resistance profiling, lineage typing, and cluster analysis (Ghodousi et al., 2025; Castro-Rodriguez et al., 2024; Meehan et al., 2019; Kremer et al., 2005, 1999; Supply et al., 2006, 2001). WGS provides a powerful tool for accurate TB diagnosis, drug resistance analysis, molecular epidemiological surveillance, and formulation of TB prevention and control strategies (Kremer et al., 1999; Supply et al., 2006, 2001; Xu et al., 2025; Rajasekaran et al., 2024).

Based on a project of the National Key Research and Development Program of China, our research team aimed to conduct in-depth studies on acid-fast positive clinical isolates from Anhui, Fujian, and Hunan Provinces. We used existing advanced technologies (including WGS). The focus was on analyzing isolate species composition, genetic lineage distribution, and drug resistance characteristic profiles, while evaluating the efficacy of WGS in predicting drug resistance. By revealing the molecular characteristics of strains from different regions, this study intends to deepen the understanding of *Mycobacterium* and provide a scientific basis for formulating more precise regional TB prevention and control strategies.

## Materials and methods

### Strain source

This cross-sectional study investigated acid-fast bacillus (AFB) smear-positive isolates collected in 2024 from tuberculosis (TB) designated medical institutions in Anhui, Hunan, and Fujian Provinces. The original strains were cultured to the second generation for WGS and DST. Meanwhile, detailed demographic and clinical data of the corresponding patients were systematically collected. These included but were not limited to age, gender, previous treatment history, and clinical manifestations.

### DST

For strains identified as MTB via molecular biology, phenotypic DST against 12 anti-TB drugs (Table 1) was performed using the Sensititre™ MYCOTBI Minimum Inhibitory Concentration (MIC) Plate (Trek Diagnostic Systems, Cleveland, OH, USA).

**Table 1:** Composition and critical concentrations of 12 anti-TB drugs.

Anti-TB drug	Concentration (ug/ml)	Critical concentration (ug/ml)
Isoniazid (INH)	0.03–4	0.2
Rifampicin (RIF)	0.12–16	1
Ethambutol (EMB)	0.5–32	5
Amikacin (AMK)	0.12–16	4
Ethionamide (ETH)	0.3–40	5
Kanamycin (KAN)	0.6–40	5
Moxifloxacin (MXF)	0.06–8	0.5
Ofloxacin (OFLX)	0.25–32	2
Para-aminosalicylic acid (PAS)	0.5–64	2
Rifabutin (RFB)	0.12–16	0.5
Streptomycin (STR)	0.25–32	2
Cycloserine (CSC)	2–256	25

### DNA extraction and quality control

Genomic DNA of *Mycobacterium* was extracted via the Cetyltrimethylammonium Bromide (CTAB) method (Xu et al., 2025). Nanodrop ND-1000 Nucleic Acid Analyzer was used to detect DNA purity, 1% agarose gel verified DNA integrity. The Qubit system quantified DNA concentration.

### WGS

DNA samples of strains passing library construction were sequenced on the Illumina NovaSeq platform using the paired-end 150 (PE150) mode, with a planned sequencing depth of 500X.

### Data QC

FastQC (v0.23.2; Rajasekaran et al., 2024) was used for quality control of raw sequencing data. Low-quality reads filtered out. The final analytical data required a base proportion of ≥99% at the Q30 level.

### Drug resistance and lineage analysis

Kmerfinder (v1.4.18; Jensen et al., 2021) software was used for species identification. Drug resistance-associated mutations and strain lineages were identified using TB-Profiler (v6.6.0; Phelan et al., 2019). Genotypic resistance was determined according to the WHO Catalog of mutations in the *Mycobacterium tuberculosis* complex (second edition, 2023; World Health Organization, 2026).

### Phylogenetic analysis

Subsequently, software including Bowtie2 (v2.4.4-1; Langmead and Salzberg, 2012), SAMtools (v1.13-4; Li et al., 2009), and VarScan (v2.4.6; Koboldt et al., 2009) was used to align all MTB genomes with the reference genome (NC000962.3). After obtaining whole-genome single nucleotide polymorphism (SNP) sites, SNPs located in PE/PPE genes, drug resistance-related regions, and repetitive fragments were removed to acquire core genome SNPs. These were further filtered with the following parameters: min-coverage > 20, min-reads > 2, min-avg-qual > 20, min-var-freq > 0.75, and *p* > 0.01; finally, IQ-TREE (v3.0.1) software was used to construct a phylogenetic tree. ITOL software was applied for supplementary modification.

### Statistical analysis

Microsoft Excel organized demographic/clinical data, analyzed phenotypic DST results, and calculated indicators [sensitivity, specificity, positive predictive value (PPV), negative predictive value (NPV)] of WGS-based drug resistance prediction. SPSS (v20.0.0) performed statistical analysis. The chi-square test compared inter-group rate differences, with *P* < 0.05 considered statistically significant.

## Results

### Strain collection and valid data

A total of 1,446 clinical acid-fast positive isolates were collected (400 from Anhui, 596 from Fujian, 450 from Hunan). Due to incomplete patient info for some strains, subculture failure, unqualified WGS data, or phenotypic DST contamination, 1,383valid WGS results and 884valid phenotypic DST results were finally obtained (Table 2). Discrepancies between WGS and DST datasets arose because second-line DST was not yet conducted on Hunan isolates (n=450), and some isolates lacked paired first-line DST due to contamination or insufficient culture growth.

**Table 2:** Summary table of strain completion status.

Province	Collected strains	First-line DST	Second-line DST	WGS	DST-WGS correspondence
Anhui	400	343	343	354	299
Hunan	450	391	0	450	289
Fujian	596	541	541	579	538
Total	1,446	1,275	884	1,383	1,126

### Species identification results

Among 1,383 strains included in the statistical analysis, the vast majority were MTB (1,319/1,383, 95.30%). NTM accounted for 3.40% (47/1,383, Table 3). In addition, a small number of non-Mycobacterium microorganisms (17 strains in total) were detected, including acid-fast weakly positive strains such as Nocardia farcinica (3 strains) and *Gordonia* spp. (4 strains), as well as Bacillus subtilis (6 strains). Among NTM, 82.98% were slow-growing mycobacteria (SGM), and 17.02% were rapidly growing mycobacteria (RGM).

**Table 3:** Detected species of NTM and corresponding proportions.

*Mycobacterium* spp.	Category	Hunan	Fujian	Anhui	Total	Percentage (%)
*intracellulare*	SGM	2	2	24	28	58.33
*gordonae*	SGM	0	6	0	6	12.5
*abscessus*	RGM	1	1	2	4	8.33
*kansasii*	SGM	1	0	3	4	8.33
*massiliense*	RGM	0	0	2	2	4.17
*fortuitum*	RGM	0	1	0	1	2.08
*vanbaalenii*	RGM	0	1	0	1	2.08
*coryneforme*	SGM	0	1	0	1	2.08

### Species spectrum

**Figure 1:**
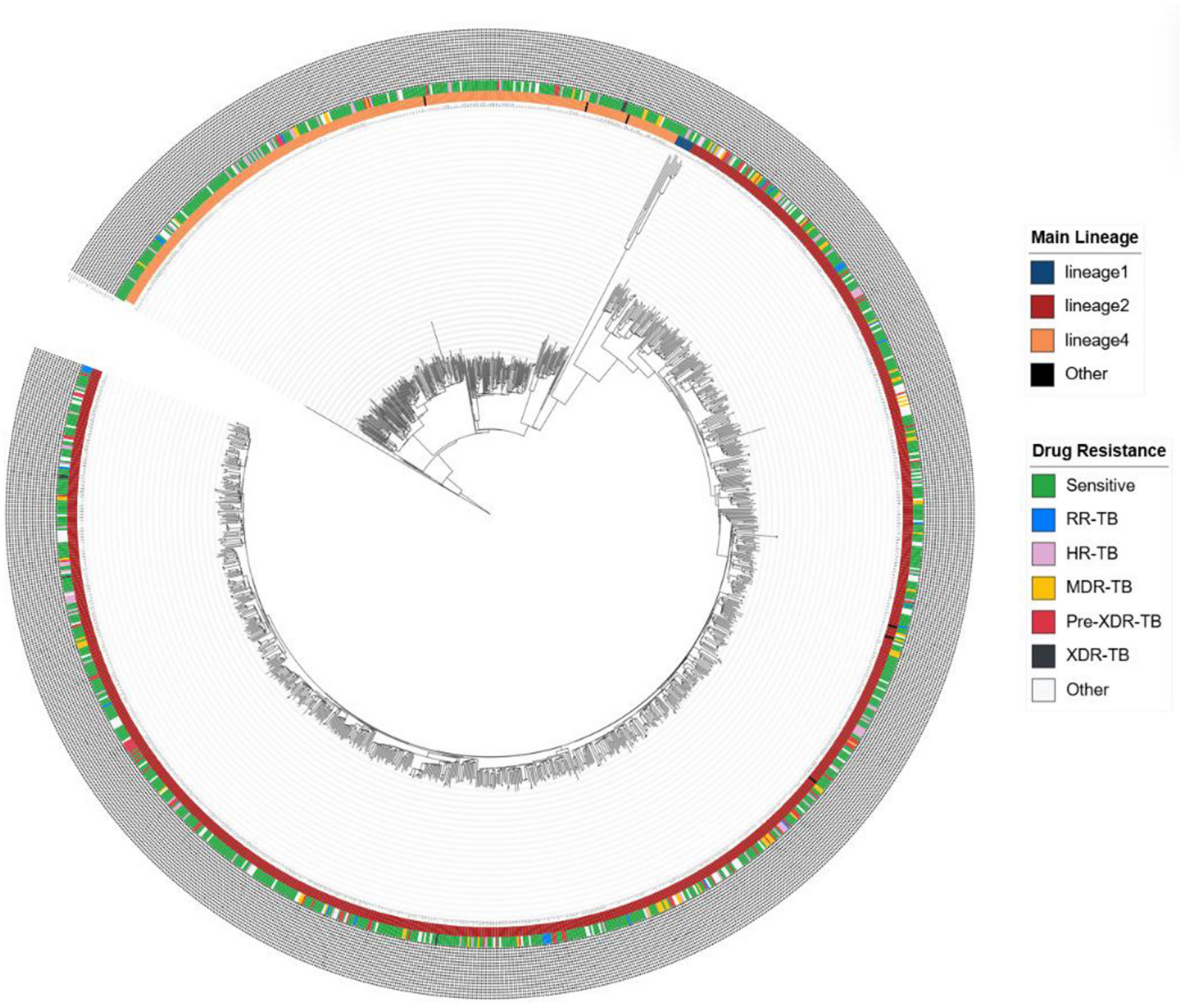
Phylogenetic analysis of 1,319 *Mycobacterium tuberculosis* clinical isolates. The innermost ring indicates lineage distribution, the middle ring categorizes resistance types, and the outermost ring displays detailed drug susceptibility profiles.

Among MTB strains, most (74.07%, 977/1,319) belonged to lineage 2, followed by lineage 4 (24.94%, 329/1,319) and lineage 1 (0.76%, 10/1,319). A small proportion (0.23%, 3/1,319) showed contamination or mixed infection, all of which were between lineage 2 and lineage 4. The dominant sublineage in lineage 2 was L2.2.1 (95.91%, 937/977). In lineage 4, the dominant sublineage was L4.5 (54.10%, 178/329), followed by L4.4.2 (27.05%, 89/329; Figure 1).

Significant inter-provincial differences were observed in the MTB lineage structure (Table 4). Lineage 2 was the dominant circulating strain in all three provinces, but its proportion showed a gradient. It was highest in Anhui Province (central China) at 90.68% (292/322), followed by Hunan Province at 73.76% (326/442), and lowest in Fujian Province (eastern China) at 66.24% (359/542). Conversely, the proportion of lineage 4 increased in the order of Anhui, Hunan, and Fujian, with proportions of 8.07%, 26.24%, and 33.76%, respectively. The overall distribution of lineage 2 and lineage 4 among the three provinces was significantly different (χ^2^ = 64.454, *p* < 0.001).

**Table 4:** MTB lineage distribution in three provinces of China.

Province	Lineage2	Lineage4	χ^2^	*P*
Anhui	292	30	64.454	*p* < 0.001
Hunan	326	116		
Fujian	359	183		

### Phenotypic drug resistance

The overall drug resistance rate was 32.08% (409/1,275), and the MDR rate was 11.14% (142/1,275). While the total drug resistance rate did not differ significantly among provinces (*p* > 0.05), the MDR rate did (*p* < 0.05), with Hunan Province having a relatively higher rate. For first-line anti-TB drugs (Table 5), INH had the highest resistance rate (19.37%), followed by STR (17.10%), RIF (13.49%), and EMB (5.49%). Only the RIF resistance rate showed a statistically significant inter-provincial difference, being higher in Hunan Province.

**Table 5:** Comparison of first-line anti-TB drug resistance among three provinces.

Drug	Resistance	Hunan	Fujian	Anhui	Total	χ^2^	*P*
INH	Yes	87	106	54	247	4.983	0.083
	No	304	435	289	1,028		
STR	Yes	79	92	47	218	5.455	0.065
	No	312	449	296	1,057		
RIF	Yes	70	65	37	172	9.680	0.008
	No	321	476	306	1,103		
EMB	Yes	25	31	14	70	1.987	0.370
	No	366	510	329	1,205		
MDR	Yes	58	52	32	142	7.803	0.020
	No	333	489	311	1,133		

As phenotypic DST for isolates from Hunan Province has not yet been fully completed, the analysis of second-line anti-tuberculosis drugs was performed only on isolates from Anhui and Fujian Provinces. Analysis of second-line anti-TB drugs for 884 strains from Fujian and Anhui Provinces (Table 6) showed that fluoroquinolones (FQs) had the highest resistance rates (MXF, 11.99%; OFL, 11.65%), followed by RFB (9.95%), ETH (2.51%), PAS (1.81%), KAN (1.47%), and AMI (1.02%). Resistance rates for FQs and KAN showed statistically significant differences between the two provinces (*p* < 0.05).

**Table 6:** Second-line anti-TB drug resistance comparison between two provinces.

Drug	Resistance	Fujian	Anhui	Total	χ^2^	*P*
OFL	Yes	50	53	103	7.863	0.005
	No	491	290	781		
MXF	Yes	55	51	106	4.399	0.036
	No	486	292	778		
AMI	Yes	4	5	9	1.075	0.300
	No	537	338	875		
RFB	Yes	55	33	88	0.070	0.792
	No	486	310	797		
PAS	Yes	8	8	16	0.861	0.354
	No	533	335	869		
ETH	Yes	21	11	32	0.274	0.601
	No	520	332	852		
KAN	Yes	4	9	13	5.514	0.023
	No	537	334	871		

### WGS analysis

A total of 1,126 isolates with both WGS and DST results available were included in the analysis of WGS predictive performance (Table 7). For first-line drugs, WGS demonstrated sensitivities >85% and specificities >96%. Sensitivity was highest for RIF (95.48%) and INH (91.79%). Specificity was highest for RIF (98.70%). For second-line drugs, specificity for MXF and AMK reached 100%. Sensitivity was 100% for AMK, 95.74% for ETH, and 80.20% for MXF.

**Table 7:** Accuracy of WGS-predicted drug resistance rates.

Drug	Resistant phenotype			Susceptible phenotype			Sensitivity (95% CI)	Specificity (95%)	PPV	NPV
	R	S	Total	R	S	Total				
INH	190	17	207	18	901	919	91.79 (86.96–95.00)	98.04 (96.86–98.80)	91.35 (86.46–94.65)	98.15 (96.99–98.88)
RIF	148	7	155	13	958	971	95.48 (90.56–98.01)	98.70 (97.72–99.27)	91.93 (86.30–95.45)	99.29 (98.48–99.69)
EMB	51	6	57	39	1,030	1,069	89.47 (77.81–95.65)	96.35 (95.00–97.36)	55.67 (45.82–66.94)	99.42 (98.68–99.76)
STR	159	26	185	19	922	941	85.95 (79.90–90.45)	97.98 (96.80–98.75)	89.33 (83.61–93.28)	97.26 (95.95–98.16)
MXF	81	20	101	0	736	736	80.20 (70.84–87.21)	100.00 (99.35–100.00)	100.00 (94.36–100.00)	97.35 (95.87–98.33)
AMI	7	0	7	0	830	830	100.00 (56.09–100.00)	100.00 (99.43–100.00)	100.00 (56.09–100.00)	100.00 (99.43–100.00)
ETH	45	2	47	18	772	790	95.74 (84.27–99.26)	97.72 (96.35–98.60)	71.43 (58.47–81.76)	99.74 (98.96–99.96)
KAN	7	2	9	1	827	828	77.78 (40.19–96.05)	99.88 (99.22–99.99)	87.50 (46.68–99.34)	99.76 (99.03–99.96)

### Concordance analysis

For the eight anti-TB drugs, the overall concordance between genotypic prediction and phenotypic DST results was 86.86% (978/1,126). Discordance was observed in 148 strains (Table 8). Among these, two types of discordance were notable. First, 50 isolates were phenotypically resistant but genotypically susceptible (wild-type, WT), carried no known resistance-associated mutations. Second, for RIF specifically, 26 isolates were phenotypically susceptible but genotypically resistant. These carried *rpoB* mutations such as Leu430Pro, His445Asn, etc., which are classified as borderline mutations.

**Table 8:** Discordance between genotypic and phenotypic drug susceptibility.

Drug	Gene	Mutation	Phenotypic DST	Genotypic WGS	Isolates	WHO_Group
INH						
	*inhA*	−777C>T	S	R	11	Group1
		−154G>A	S	R	5	Group1
	*katG*	Lle462Thr	S	R	1	–
	*inhA* + *katG*	−777C>T + Gly285Asp	S	R	1	Group1+ Group3
		−154G>A + Trp191Arg	S	R	1	Group1+ Group3
	*ahpC*	−48G>A	R	S	2	Group3
		−81C>T	R	S	1	Group3
	*ahpC*+*KatG*	−52C>T + Gly285Asp	R	S	4	Group3+ Group3
		−81C>T + Thr275Pro	R	S	1	Group3+ Group3
	WT	–	R	S	8	
RIF						
	*rpoB*	Leu430Pro	S	R	8	Group1
		His445Asn	S	R	8	Group1
		Leu452Pro	S	R	7	Group1
		His445Leu	S	R	1	Group1
		Leu430Pro + Met434Leu	S	R	1	Group1+ Group2
	WT	–	R	S	3	
EMB						
	*embB*	Met306lle	S	R	16	Group1
		Met306Val	S	R	13	Group1
		Gly406Ala	S	R	6	Group1
		Gly406Asp	S	R	4	Group1
		GIy406Ser	S	R	1	Group1
		GIn497Lys	S	R	2	Group1
		Gln497Arg	S	R	2	Group1
		Asp328Tyr	S	R	1	Group3
	*embA*+*embB*	−16C>T + Met306Ile	S	R	1	Group3+ Group1
	*embA*	−12C>T	R	S^*^	1	Group1
	WT	–	R	S	4	
STR						
	*rpsL*	Lys43Arg	S	R	7	Group1
		Lys88Arg	S	R	1	Group1
	*gid*	102delG	S	R	4	–
		273dupA	S	R	2	–
		115delC	S	R	1	–
	*rrs*	514A>C	S	R	3	Group1
	*rpsL*	Lys43Thr	R	S	2	Group3
		Lys88Thr	R	S	1	Group3
	*gid*	Pro75Arg	R	S^*^	1	Group3
		Leu108Arg	R	S	1	Group3
		Leu79Ser	R	S	1	Group3
		Trp148^*^	R	S	1	Group3
	*rrs*	514A>T	R	S	2	Group3
	WT	–	R	S	15	
	*gyrB*	Asp461Asn	S	R^*^	1	Group2
	*gyrA*	Asp94Val	R	S^*^	2	Group3
	WT	–	R	S	18	
KAN						
	*eis*	−12C>T	S	R	1	Group1
		−10G>A	R	S	1	Group3
		−8C>A	R	S	1	Group3
ETH						
	*inhA*	−777C>T	S	R	6	Group1
		−154G>A	S	R	3	Group2
		−770T>C	S	R	1	Group2
		−779G>T	S	R	2	Group2
		−154G>A + Ile194Thr	S	R	1	Group2+ Group3
		−777C>T + Ser94Ala	S	R	2	Group1+ Group2
		−777C>T + Ile21Thr	S	R	2	Group1+ Group3
	*ethA*	11dupA	S	R	2	–
		1353dupG	S	R	1	–
		479delC	S	R	1	–
		1047delT	S	R	1	–
		Trp228^*^	S	R	1	Group2
		Tyr382^*^	S	R	1	Group2
		762_765dupGAAG	S	R	1	–
	*ethA* + *inhA*	775dupC +−777C>T	S	R	1	–+ Group1
	WT	–	R	S	2	

**Figure 2:**
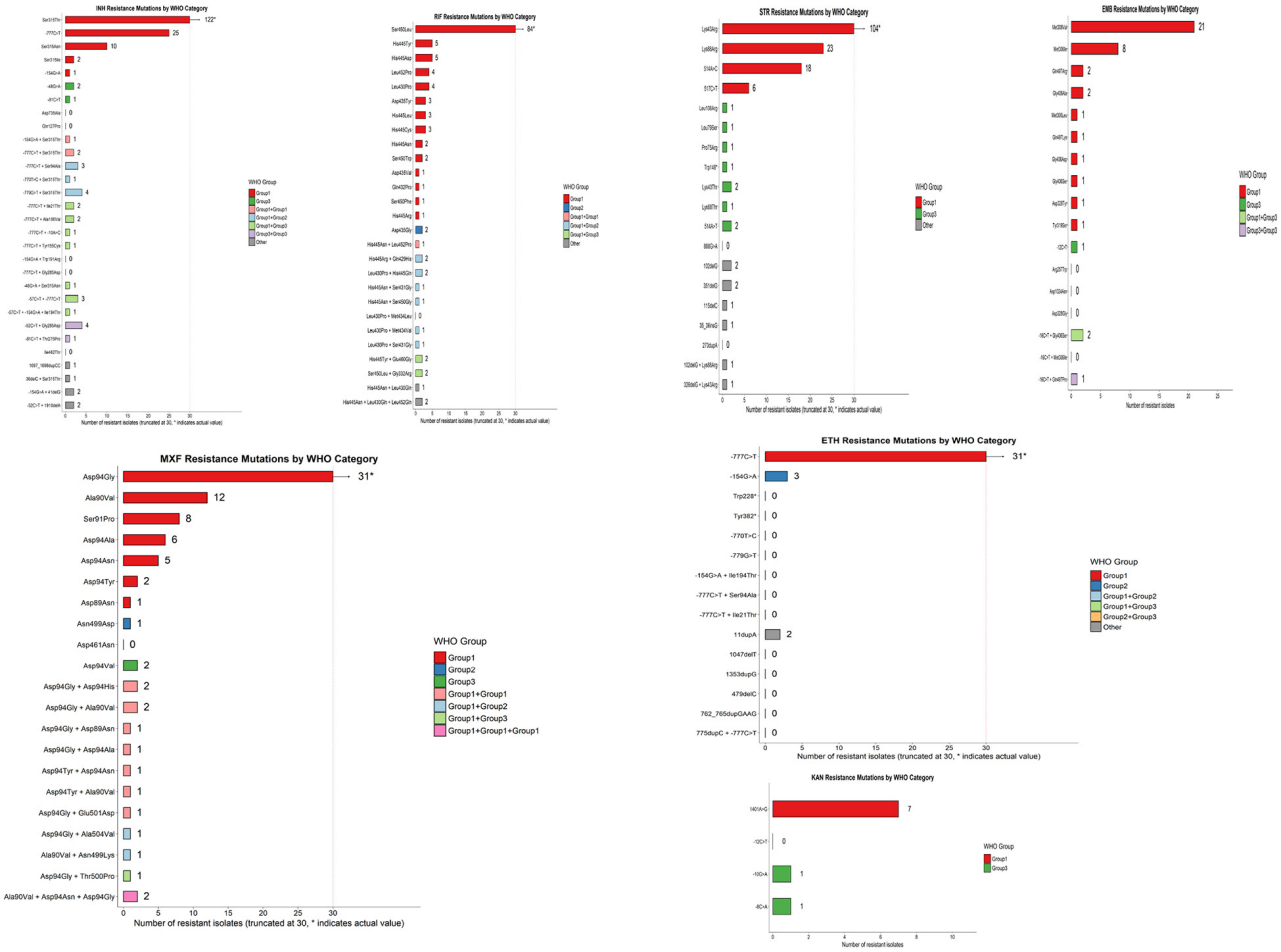
Profiles of resistance-associated mutations for seven anti-tuberculosis drugs.

For INH, the most frequently observed resistance mutation was *katG* Ser315Thr (122 isolates). RIF resistance was predominantly conferred by *rpoB* Ser450Leu (84 isolates). STR resistance was most associated with *rpsL* Lys43Arg (104 isolates). All these substantially outnumbered other mutation types. Ethambutol resistance was primarily driven by mutations at codon 306 of *embB*, particularly Met306Val (21 isolates). For moxifloxacin, the predominant resistance mutation was *gyrA* A549Gly (31 isolates). Overall, the resistance-associated mutations identified for each drug showed strong concordance with the WHO mutation catalog grading system (Figure 2).

## Discussion

Based on large-scale WGS, this study systematically revealed the species distribution, genetic lineage, and drug resistance characteristics of *Mycobacterium* in Anhui, Fujian, and Hunan Provinces, China. The results indicate that MTB accounted for 95.30% of acid-fast positive samples, while NTM accounted for 3.40%. This proportion is significantly lower than that reported in a national meta-analysis covering 27 provinces/municipalities/autonomous regions of China by Wang et al. (2021). That meta-analysis indicated that the pooled proportion of NTM among *Mycobacterium* was 12% (11.2%−12.7%), reaching 12.7% (11.3%−14.2%) in initial clinical samples. Even when compared with previous reports from East China and Central China (the regions where the three provinces are located), the proportion of NTM in this study remained at a relatively low level. For example, an early report (Bao et al., 2020) from Anhui Province showed an NTM isolation rate of approximately 4.0%. In some areas of Hunan Province, the NTM proportion once reached 10.2% (Chen et al., 2019).

This discrepancy may be attributed to multiple factors. From the perspective of detection technology, WGS has higher accuracy in species identification than traditional PNB/TCH differential media and single 16S rDNA sequencing. This reduces the misclassification between NTM and MTB. From the perspective of sample background, the samples in this study focused more on confirmed active TB cases rather than suspected cases. This may also lower the detection rate of NTM. These findings suggest that MTB—with well-defined pathogenicity—remains the dominant pathogen of *Mycobacterium* infection in Anhui, Fujian, and Hunan Provinces. However, due to the convenience sampling strategy and the focus on confirmed TB patients, the NTM proportion of 3.40% should be interpreted as a minimum estimate rather than a definitive regional prevalence rate. Among NTM, 82.98% were slow-growing mycobacteria (SGM), mainly *Mycobacterium intracellulare*, and 17.02% were RGM, mainly *Mycobacterium abscessus*—a distribution consistent with the epidemiological trend of NTM in China (Liu et al., 2021). Therefore, further efforts are needed to advance the etiological diagnosis of *Mycobacterium* species.

The genetic lineages of MTB were dominated by lineage 2 (74.07%) and lineage 4 (24.94%). This is consistent with the characteristics of the main circulating MTB strains in China. There were differences in lineage structure among the three provinces. This indicates that the genetic background of MTB in East to Central China is not fully homogeneous. Instead, it exhibits the characteristics of “conservative dominance of the core lineage (L2) and heterogeneous differentiation of inter-provincial structure.” The dominant status of L2 in the three provinces confirms the commonality in the genetic origin of MTB in China. Meanwhile, the gradient differences in lineage proportions among provinces reflect the specificity of MTB transmission dynamics and evolutionary directions in different provinces. The overall drug resistance rate was 32.08%, and the MDR rate was 11.14%. Both are higher than the national average (7.5%; Wang et al., 2012). This may be related to the sample source, leading to a certain degree of selection bias. For first-line anti-TB drugs, INH and STR had relatively high resistance rates. Their molecular mechanisms were highly consistent with the *katG* S315T mutation, *inhA* promoter mutation, and *rpsL* K43R mutation (Pinhata et al., 2021). For second-line drugs, the resistance rate to FQs was approximately 12%, with significant differences between Fujian and Anhui Provinces. Although China has achieved the TB control target set for 2025, the high drug resistance rates in these provinces suggest that the prevention and control efforts—especially for the drug-resistant TB—need to be further strengthened.

WGS showed excellent predictive sensitivity (>91%) and specificity (>98%) for RIF and INH. This confirms that mutations such as *KatG* S315T and *rpoB* S450L can serve as core drug resistance markers, suitable for rapid clinical screening. Two distinct types of discordance were observed. The first type involved 50 wild-type strains that were phenotypically resistant but lacked any known resistance mutations. Their resistance mechanisms remain completely unknown, highlighting a major gap in current mutation databases. The second type involved 26 RIF isolates that were phenotypically susceptible but carried borderline *rpoB* mutations (e.g., Leu430Pro, His445Asn). Borderline mutations are known to mediate only low-level or conditional resistance, frequently causing false-positive genotypic predictions (Van Deun et al., 2009, 2021). These two types of discordance should not be confused; the former suggests undiscovered mechanisms, while the latter reflects known limitations of phenotypic DST at low drug concentrations. For second-line drugs, the predictive accuracy of WGS for AMI resistance reached 100%. However, the sensitivity for MXF was relatively low (80.20%), and the PPV for EMB and ETH were relatively low. Additionally, some strains were phenotypically resistant but genotypically susceptible. This is consistent with previous findings (Pei et al., 2024) that 45.8% of such strains carry efflux pump gene mutations. Moreover, the insufficient coverage of insertion/deletion (indel) mutations in the WHO Mutation Catalog may also limit the prediction efficacy for some drugs. Nevertheless, WGS is more efficient than traditional DST. It can serve as a core tool for drug resistance screening and molecular epidemiological surveillance, despite the limitations of existing mutation catalogs in predicting resistance to certain drugs. This highlights that the application of resistance-associated mutations should be tailored to locally prevalent strains by prioritizing dominant mutation combinations.

This study had several limitations. First, the lack of phenotypic DST for second-line drugs in Hunan Province limited the comprehensiveness of comparative analysis. Second, some drugs were excluded from the evaluation due to low reliability of phenotypic results (e.g., CYC, PAS; Shi, 2021; Barrera et al., 2008) or incomplete mutation information (e.g., RFB, OFL). Third, the statistical power was limited by the low frequency of certain mutations (e.g., only 7 strains were resistant to AMI). Additionally, the isolates were collected from only three provinces (Anhui, Fujian, Hunan) using a convenience sampling strategy. While this provides valuable regional insights, the findings—particularly the observed drug resistance rates and lineage distributions—may not be fully representative of the broader Chinese population or generalizable to other regions with different socioeconomic and epidemiological backgrounds. This geographical limitation, combined with the limited demographic data available, raises a concern regarding potential sampling bias.

However, this study also had notable strengths. It covered three provinces (Anhui, Fujian, Hunan) and included 1,383 clinical isolates. The large sample size and wide geographical coverage ensured strong representativeness and statistical power to reflect the epidemiological characteristics of *Mycobacterium* in Central and East China. Compared with traditional identification methods, WGS significantly improved the accuracy and resolution of species identification. It showed particular advantages in the accurate identification of NTM. Furthermore, this study systematically evaluated the predictive sensitivity, specificity, PPV, and NPV of WGS for first- and second-line anti-TB drugs. This provides solid data support for the application of WGS in clinical drug resistance screening. The finding that Hunan Province had significantly higher MDR and RIF resistance rates than the other two provinces has important guiding significance for formulating regional TB prevention and control strategies. Additionally, the identification of 50 WT strains that were phenotypically resistant suggests the existence of undiscovered drug resistance mechanisms. These points out directions for subsequent mechanistic studies. Finally, full-process quality control and cross-validation using multiple software were implemented throughout the workflow (from DNA extraction, library construction, and sequencing to bioinformatics analysis). This ensured data reliability and the scientificity of the analysis results.

In conclusion, based on WGS technology, this study systematically revealed the species distribution, genetic lineage, and drug resistance characteristics of clinically isolated MTBC from Anhui, Fujian, and Hunan Provinces. The relatively low detection rate of NTM (3.40%) should be considered a minimum estimate. Continuous etiological surveillance is necessary to avoid misdiagnosis and inappropriate treatment. Furthermore, the ongoing burden of drug-resistant TB highlights the need for strengthened prevention and control efforts. Particular emphasis should be placed on improving patient management and treatment adherence to curb transmission. Notably, the presence of 50 phenotypically resistant but genotypically susceptible strains suggests that additional resistance mechanisms remain to be elucidated. This warrants further investigation to expand current mutation databases. Collectively, these findings provide important molecular epidemiological evidence to inform precision prevention and control strategies as well as personalized treatment for TB in China. Future large-scale, prospective multicenter studies integrating genomic and clinical data are recommended to further advance these efforts.

## Data Availability

The datasets presented in this study can be found in online repositories. The names of the repository/repositories and accession number(s) can be found at: https://www.ncbi.nlm.nih.gov/, BioProject ID: PRJNA1397020.
